# A Rare Case of Parvimonas micra Bacteremia in a 90-Year-Old Male With Severe Sepsis and Multifocal Pneumonia: A Case Report

**DOI:** 10.7759/cureus.93198

**Published:** 2025-09-25

**Authors:** Rishi S Ada, Shreya Kolli, Samuel George, Brice A Bulotovich, Moulali Shaik

**Affiliations:** 1 Medicine, Texas Tech University Health Sciences Center El Paso Paul L. Foster School of Medicine, El Paso, USA; 2 Internal Medicine, Texas Tech University Health Sciences Center El Paso Paul L. Foster School of Medicine, El Paso, USA

**Keywords:** anaerobic bacteremia, elderly sepsis, gram-positive cocci, maldi-tof, multifocal pneumonia, parvimonas micra, rare bloodstream infection, sepsis diagnostics, targeted antibiotic therapy, urinary tract infection

## Abstract

*Parvimonas micra* (*P. micra*) is an anaerobic, gram-positive coccus that is part of the normal flora of the oral cavity and gastrointestinal tract, but is rarely implicated in systemic infections. We present a case of a 90-year-old male with severe sepsis, multifocal pneumonia, and a urinary tract infection, whose blood cultures revealed *P. micra* bacteremia, an extremely rare infection. The diagnosis was made using rapid matrix-assisted laser desorption ionization-time of flight (MALDI-TOF) technology, which enabled targeted antibiotic therapy. This case emphasizes the importance of considering rare anaerobic pathogens in immunocompromised or elderly patients with sepsis, and highlights the role of advanced microbiological diagnostics in early detection and optimized medical management.

## Introduction

*Parvimonas micra *(*P. micra*), a gram-positive, obligate anaerobic coccus, is a common commensal organism in the oral cavity, gastrointestinal tract, and female genitourinary system [1]. It has traditionally been associated with oral and dental infections, including periodontal disease, osteomyelitis, and abscesses [1]. However, its role as a pathogen in systemic infections is rarely reported, and only a handful of cases of *P. micra* bacteremia exist in the medical literature [2]. Diagnostic confirmation of *P. micra* is challenging due to its anaerobic nature, which limits recovery on routine cultures. In recent years, rapid identification techniques such as matrix-assisted laser desorption ionization-time of flight (MALDI-TOF) mass spectrometry and 16S rRNA sequencing have become increasingly available, allowing more timely and accurate recognition of anaerobic bacteremia. These tools are particularly valuable in centers where access to specialized anaerobic culture facilities is limited. 

Rarity and clinical relevance of *Parvimonas micra* bacteremia

Most anaerobic bacteremia cases are caused by *Bacteroides*, *Fusobacterium*, and *Clostridium* species. *Parvimonas micra* is an infrequent but emerging anaerobic pathogen in bloodstream infections [3]. A retrospective review of 25 cases in Japan found that *P. micra* bacteremia was frequently associated with dental infections, gastrointestinal malignancies, or prosthetic infections, but its presence in patients without these risk factors is extremely rare [1].

A systematic literature review identified only 30 published cases of *P. micra* infections. A 2022 case report and systematic review explored the routes of infection for *P. micra* and its association with brain abscesses, septic arthritis, and endocarditis, emphasizing the importance of early recognition and targeted therapy [1].

Pathogenesis and diagnostic challenges

Due to its anaerobic nature, *P. micra* is difficult to culture using standard microbiological techniques [3]. Traditional blood cultures may fail to detect anaerobic organisms, leading to delays in diagnosis and suboptimal antimicrobial therapy. The introduction of MALDI-TOF mass spectrometry has significantly improved the rapid identification of anaerobic organisms, including *P. micra*, ultimately leading to better patient outcomes [4].

Clinical significance of this case

The case described here presents one of the first reports of *P. micra* bacteremia in an elderly man with multifocal pneumonia and sepsis, without a clear primary source of infection or the typical aforementioned risk factors. This highlights the potential for *P. micra* to cause systemic infection in immunocompromised or elderly patients, even in the absence of obvious risk factors. Given the limited number of reported cases, this case adds to the growing body of literature demonstrating *P. micra’s* potential role as an emerging anaerobic pathogen and the importance of MALDI-TOF technology for early detection.

## Case presentation

Patient description

A 90-year-old male with a past medical history of hypertension, type 2 diabetes mellitus, chronic kidney disease (stage 3), chronic hypoxemic respiratory failure, bronchiectasis, prostate cancer, and congestive heart failure was transferred to our hospital from a community emergency department for further evaluation of severe sepsis, multifocal pneumonia, and pleural effusion.

Case history

The patient initially presented to the community emergency department with generalized weakness, shortness of breath, and a productive cough of three days' duration. He reported that he woke up unable to get out of bed due to severe fatigue and dyspnea. He also reported low-grade fever, body aches, and malaise but denied chest pain, nausea, or vomiting. He was diagnosed with sepsis due to pneumonia and a urinary tract infection, and was started on empiric intravenous (IV) meropenem before being transferred to our facility.

Physical examination and initial workup

**Figure 1:**
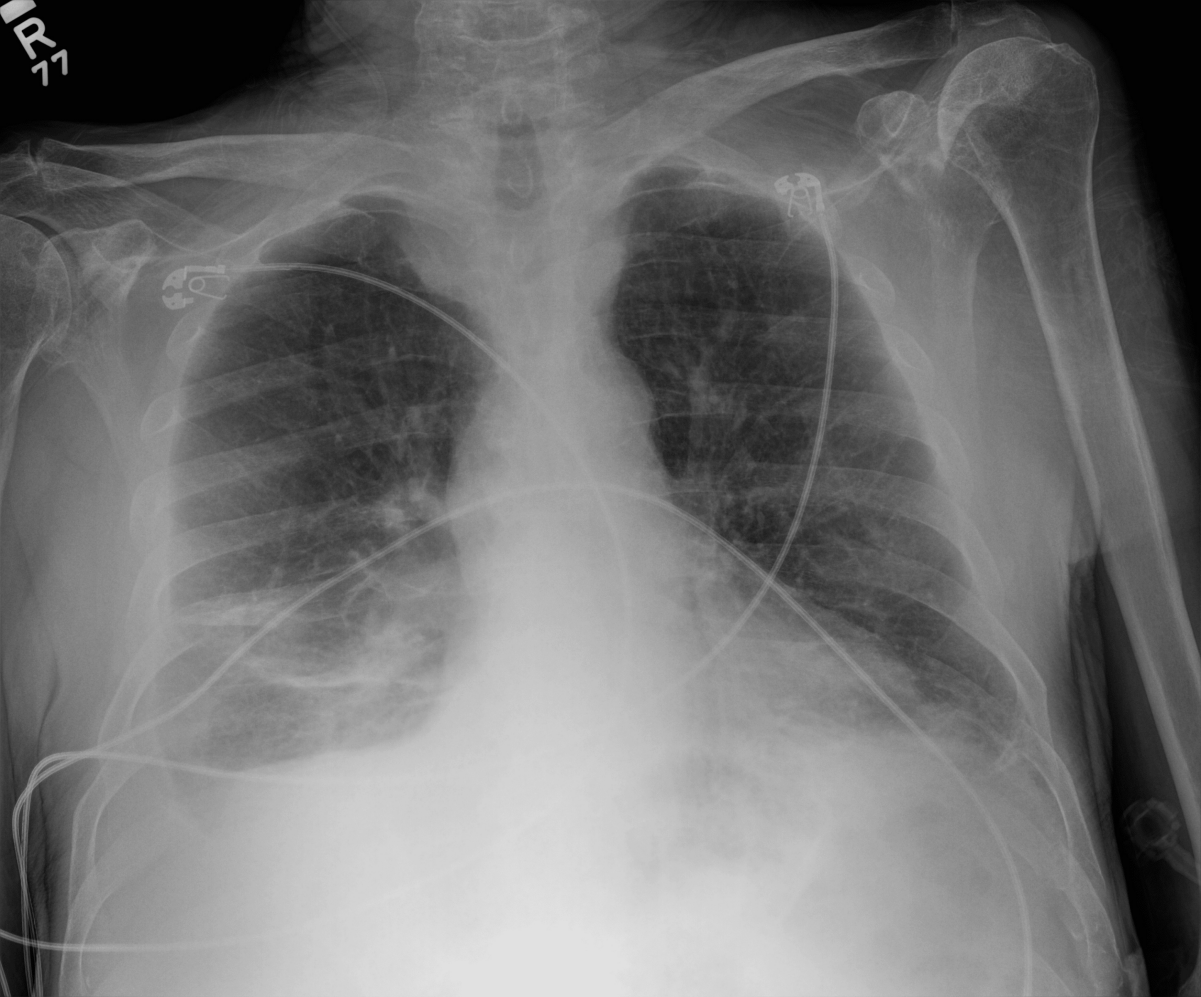
Chest x-ray showing increased opacity in the right lower lobe

The patient’s temperature on admission was 38.2 °C with a heart rate of 100 bpm and oxygen saturation of 96% on 2 L. He was found to have bilateral rhonchi, wheezing, and rales on physical examination. His labs showed leukocytosis with a white blood cell count of 15.4 (Table 1). His chest x-ray showed right lower lobe pneumonia with mild pleural effusion (Figure 1). 

**Table 1:** Relevant laboratory values on admission WBC, white blood cells; BUN, blood urea nitrogen; eGFR, estimated glomerular filtration rate.

Component	Value	Reference Range	Date
WBC	15.4 (H)	4.5-11.0 x10^3^/µL	1/11/25
Procalcitonin	<0.04	<0.10 ng/mL	1/11/25
Creatinine	1.55 (H)	0.6-1.3 mg/dL	1/11/25
BUN	34 (H)	7-20 mg/dL	1/11/25
eGFR	42.3	>60 mL/min/1.73 m²	1/11/25

Two blood culture sets were obtained as per sepsis protocol, and one of the two was positive. The blood culture was found to grow gram-positive cocci in anaerobic bottles (final identification: *P. micra*). The patient’s bacteremia was confirmed using an anaerobic blood culture bottle, with final identification achieved through MALDI-TOF mass spectrometry. This advanced diagnostic method was essential for the timely recognition of *P. micra*, as traditional culture methods may fail to identify anaerobes. However, MALDI-TOF is not universally available in all centers, which can limit early detection of such rare pathogens. 

Treatment and hospital course

Empiric therapy was initiated with IV meropenem for broad anaerobic and gram-negative coverage, along with IV fluids for sepsis management. Following consultation with the infectious disease team, antibiotic therapy was modified based on microbial susceptibility patterns. The team recommended continuing IV meropenem and adding IV vancomycin and metronidazole. Over the following days, the patient’s fever resolved, white blood cell count returned to baseline, and respiratory symptoms improved. The patient was discharged home with the infectious disease team’s recommendations.

## Discussion

*Parvimonas micra* is a rare cause of bacteremia, with a few cases reported in the literature. As a gram-positive, obligate anaerobic coccus, it is primarily found in the oral cavity, gastrointestinal tract, and genitourinary system. Though it is typically a commensal organism, emerging evidence suggests its ability to cause serious systemic infections, including bacteremia, endocarditis, hepatic abscesses, and osteomyelitis [2,5]. The present case of *P. micra* bacteremia in an elderly male with multifocal pneumonia and sepsis adds to the limited body of knowledge on this rare pathogen. Most reported cases have been associated with dental infections, colonic malignancies, or other defined entry points. Our patient had no known risk factors mentioned in the earlier studies, highlighting the possibility that *P. micra* can disseminate through unidentified or overlooked routes.

Recent studies suggest that *P. micra* may have greater pathogenic potential than previously thought. One such study described a patient who developed a brain abscess due to *P. micra*, despite having no identifiable primary infection site [6]. This finding parallels our case, in which *P. micra* was isolated from blood cultures without a clear source of infection. Given that this pathogen is difficult to culture using standard microbiological methods, it is likely that cases of *P. micra* bacteremia could be underreported or misattributed to other causes of sepsis. The introduction of MALDI-TOF mass spectrometry has greatly improved the ability to rapidly identify anaerobic organisms, including *P. micra*, leading to better diagnostic accuracy and more targeted antimicrobial therapy [3]. In our case, MALDI-TOF was the key diagnostic method that allowed for rapid organism identification and guided targeted therapy. This technology has distinct advantages over conventional anaerobic cultures, which are slow, technically demanding, and often yield false negatives. However, limited access to MALDI-TOF in smaller or resource-constrained centers remains a challenge and may contribute to delays in recognition of *P. micra* infections.

The identification of *P. micra* bacteremia in this patient also raises important clinical considerations regarding anaerobic bloodstream infections. Typically, *P. micra* has been associated with polymicrobial infections, particularly in synergy with other anaerobic bacteria such as *Fusobacterium* and *Bacteroides* species [1]. However, in our patient,* P. micra* was isolated as a single organism, suggesting that it may have the potential for independent pathogenicity in vulnerable individuals. Given this patient’s advanced age, chronic comorbidities, and immunosuppressive state, it is possible that these factors facilitated hematogenous spread in the absence of a clear primary source. In terms of management, *P. micra* is generally susceptible to β-lactams, metronidazole, and carbapenems, which guided our therapeutic approach. Our patient demonstrated clinical improvement with resolution of fever and normalization of white blood cell count following targeted therapy, ultimately allowing for safe discharge home. This highlights the importance of tailoring antibiotic regimens based on susceptibility patterns to optimize outcomes in elderly patients with anaerobic bacteremia. 

The diagnostic and therapeutic challenges of *P. micra* bacteremia highlight the need for increased awareness among clinicians. Traditional blood culture methods often fail to detect strict anaerobes, leading to delayed or missed diagnoses. Additionally, empirical antibiotic regimens for sepsis may not always provide adequate anaerobic coverage, particularly if *P. micra* is not initially suspected. In our case, targeted de-escalation was guided by microbiological findings, emphasizing the importance of early pathogen identification.

Compared to existing literature, this case demonstrates that *P. micra* can cause bacteremia in the absence of classical risk factors such as recent dental procedures, gastrointestinal malignancy, or prosthetic implants. Since *P. micra* is often part of polymicrobial infections, its isolation as the sole pathogen in this case suggests it may be more virulent than previously thought. Additionally, more reports describing *P. micra-*related brain abscesses reinforce the notion that this organism can disseminate hematogenously, leading to deep-seated infections.

## Conclusions

*Parvimonas micra* bacteremia remains an exceedingly rare but potentially underdiagnosed entity. This case underscores the importance of considering anaerobic pathogens in elderly and immunocompromised patients presenting with sepsis, even when a clear infectious source is not identified. The use of MALDI-TOF mass spectrometry was critical in rapidly identifying the pathogen, allowing for the timely initiation of targeted therapy. Given the increasing use of advanced diagnostic tools, it is likely that more cases of *P. micra* bacteremia will be recognized in the future.

As more cases are reported, further research is needed to better understand the full clinical spectrum of *P. micra* infections, their risk factors, and optimal treatment strategies. Timely recognition and appropriate antimicrobial therapy remain key to improving outcomes in these rare but serious infections.
